# Evaluating the reliability of the lateral femoral condyle measuring methods by different modalities for patients with lateral patellar dislocation

**DOI:** 10.1186/s12891-024-07495-x

**Published:** 2024-05-18

**Authors:** Yunlong Zhou, Anqi Yu, Xiaoan Wu, Jinjiang Yao, Hao Tan, Huaao Wang, Chengjie Lian, Aiguo Zhou

**Affiliations:** 1 Department of Orthopaedics, the First Affiliated Hospital of Chongqing Medical University, Chongqing, 400016 China; 2 Orthopedic laboratory of Chongqing Medical University, Chongqing, 400016 China

**Keywords:** Patellar dislocation, Patellar femoral condyle index, CT, MRI, Conventional radiograph

## Abstract

**Background:**

A variety of measurement methods and imaging modalities are in use to quantify the morphology of lateral femoral condyle (LFC), but the most reliable method remains elusive in patients with lateral patellar dislocation (LPD). The purpose of this study was to determine the intra- and inter-observer reliability of different measurement methods for evaluating the morphology of LFC on different imaging modalities in patients with LPD.

**Methods:**

Seventy-three patients with LPD were included. Four parameters for quantifying the morphology of LFC were retrospectively measured by three observers on MRI, sagittal CT image, conventional radiograph (CR), and three-dimensional CT (3D–CT). The intra-class correlation coefficient was calculated to determine the intra- and inter-observer reliability. Bland–Altman analysis was conducted to identify the bias between observers.

**Results:**

The lateral femoral condyle index (LFCI) showed better intra- and inter-observer reliability on MRI and 3D–CT than on CR and sagittal CT images. The mean difference in the LFCI between observers was lowest on 3D–CT (0.047), higher on MRI (0.053), and highest on sagittal CT images (0.062). The LFCI was associated with the lateral femoral condyle ratio (ρ = 0.422, *P* = 0.022), lateral condyle index (*r* = 0.413, *P* = 0.037), and lateral femoral condyle distance (*r* = 0.459, *P* = 0.014). The LFCI could be reliably measured by MRI and 3D–CT.

**Conclusion:**

The LFCI could be reliably measured by MRI and 3D–CT. The LFCI was associated with both the height and length of LFC and could serve as a comprehensive parameter for quantifying the morphology of LFC in patients with LPD.

**Supplementary Information:**

The online version contains supplementary material available at 10.1186/s12891-024-07495-x.

## Introduction

Lateral patellar dislocation (LPD) is a common sports-related disorder, and the etiology of LPD is multifactorial, such as skeletal immaturity and anatomical malformation [1, 2]. Abnormal morphology of femoral condyle, such as trochlear dysplasia, is the most frequently reported anatomic risk factor in the literature [3, 4]. Recently, clinicians have recognized that dysplasia of lateral femoral condyle (LFC) was involved in LPD. Liu et al. [5] and Zhao et al. [6] reported that shorter posterior LFC was associated with LPD. Yang et al. [7] showed that patients with LPD had both anterior and posterior LFC deformities.

A variety of measurement methods and imaging modalities are in use to quantify the morphology of LFC, but the most reliable method has not been identified. Biedert et al. [8] reported that lateral condyle index (LCI) measured on sagittal magnetic resonance imaging (MRI) plane could reflect the height of LFC in patients with LPD. Lateral femoral condyle ratio (LFCR) serves as a measurement on conventional radiograph (CR) to assess the length of femoral condyles [9]. Lateral femoral condyle distance (LFCD) measured by transverse computed tomography (CT) or MRI images was used to evaluate the length of LFC as well [6, 10, 11]. Hodel et al. [12] and Vasta et al. [13] elucidated that lateral femoral condyle index (LFCI) measured on sagittal MRI plane or CR could reliably evaluate the morphology of LFC.

On the other hand, consensus has not been reached regarding the best imaging modality for evaluating the morphology of LFC. Each of MRI, CT, and CR has advantages, such as excellent resolution of cartilage [14], diversified image post-processing techniques [15], more convenient and cheap [16], respectively. Identifying the optimal imaging modality is important to the measurement of LFC morphology.

Given that the practicability of the measurement methods for patients with trochlear dysplasia remains elusive [17], the purpose of this study was to determine the intra- and inter-observer reliability of different measurement methods for evaluating the morphology of LFC on different imaging modalities in patients with LPD. It was hypothesized that significant variability in the measurement results between different methods and between different imaging modalities could occur. This study emphasizes the importance of accurately measuring and evaluating LFC deformities for patients with LPD, which could help orthopedist with surgical decision making and patients consultation.

## Materials and methods

### Study population

The approval had been obtained from the ethics committee before this study was started (IRB NO: 2022-K522). Patients diagnosed with LPD from 2016 to 2022 in our hospital were searched in the Electronic Medical Record System, and image data was retrospectively collected. 94 patients were considered eligible for inclusion in this study according to the inclusion criteria: patients with unilateral recurrent LPD and skeletal maturity; patients with pre-operative MRI, CR, and CT images of the affected knee joints simultaneously.

Patients who met at least one of the following criteria were excluded: patients with a history of bone fracture or surgery that may influence the reliability of the measurements (*n* = 2); patients with severe epiphysitis of the femur (*n* = 2); patients with none-standard or blurry image data (*n* = 17). The inclusion and exclusion processes were conducted by two experienced orthopedists. Consequently, 73 patients were designated as the study group in this study (showing in supplementary Fig. 1).

### MRI technique

MRI examinations were performed preoperatively by the same 1.5T MRI scanner (Siemens Magnetom Essenza, Germany). Participants were placed in a supine position with the knee being secured in the 8 channel coil. The coronal and sagittal planes were scanned with the T1-weighted turbo spin-echo (TSE) sequence and proton density (PD) TSE with the fat-suppressed (FS) sequence, and the axial plane was scanned with the PD-TSE-FS. The layer thickness was set at 4 mm, the slice gap was 0.5 mm, the field of view (FOV) was 160 mm, and the matrix size was 512 × 512. The sagittal plane of the knee joint is formed along the direction of the anterior cruciate ligament, with the scanning section parallel to the anterolateral cortex of the LFC, and there is an internal rotation of about 10–15 degrees in the anterior and posterior directions.

### CT technique

Images were obtained by a CT scanner (Siemens Somatom Perspective, Germany) in our hospital, ranging from the anterior superior iliac spine to the toes. All patients were in the supine position with the knee at full extension. The scanning parameters were as follows: tube voltage, 130 kVp; tube current 110–140 mAs; scanning layer thickness and layer spacing, both 1 mm; and matrix, 512 × 512 pixels. The field of view (FOV) varied with the individual characteristics of the patients, ranging from 220 to 450 mm.

### CR technique

The images were obtained by a CR scanner (Shimazu, Japan). The X-ray analysis included anteroposterior and true sagittal images of the knee joint with a 30° flexion and weight-bearing on one foot. The voltage was 65kvp, the current was 250 mA, and the photo distance was 100–120 cm.

### Radiological assessment

73 patients were finally enrolled in this study and were available for the radiological assessment. Three observers with different medical experience, a senior orthopedist, a junior orthopedist, and a well-trained radiologist independently assessed the morphology of LFC on MRI, CT, or CR via the picture archiving and communication system (PACS) and ADW4.6 workstation (GE healthcare, USA). All the measurements were conducted again four weeks later to assess the intra-observer agreement of each measurement, including LFCI, LFCR, LCI, and LFCD, Dejour classification. The Dejour classification system categorizes trochlear dysplasia into types A, B, C, and D based on specific imaging parameters by MRI, B-D is considered as severe trochlear dysplasia [18, 19].

### Measurements

#### LFCI

**Fig. 1:**
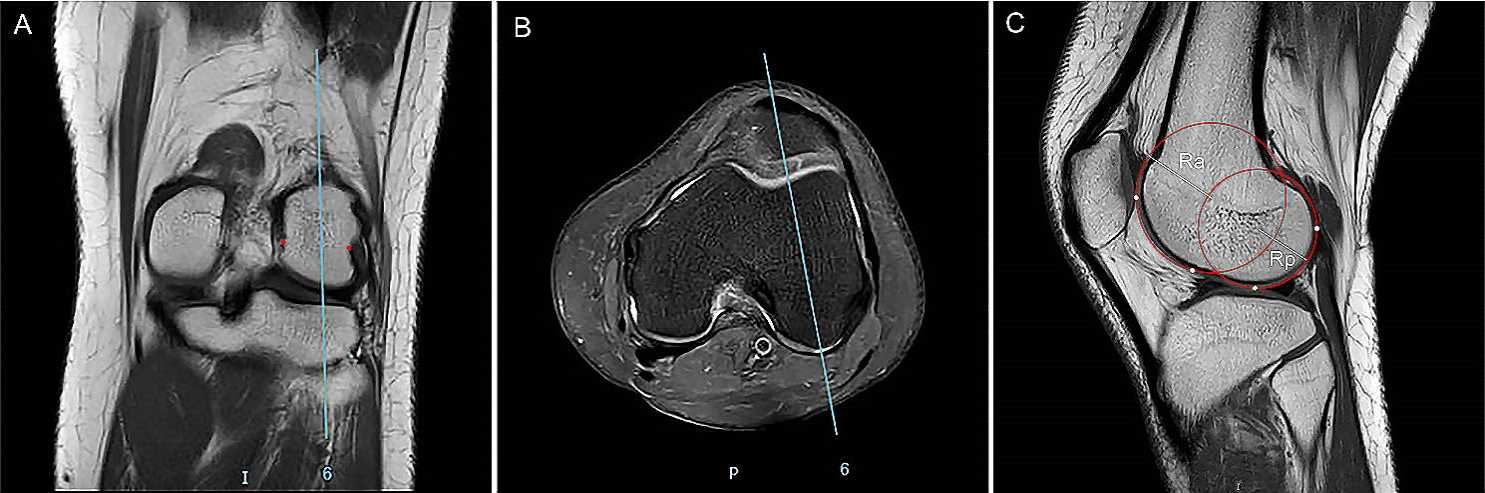
The measurement of lateral femoral condyle index (LFCI) by MRI. **(A)** MRI coronal plane with the most prominent popliteal groove is selected. **(B)** MRI axial plane showing the intact femoral trochlea and posterior femoral condyles is identified. **(C)** The relevant midsagittal plane is identified in combination with axial and coronal planes. The most inferior points of the anterior and posterior condylar cartilage, and the most anterior point and posterior point of the lateral condyle cartilage were marked (white dots). Two circles are drawn to the cartilaginous margin and pass through the marked points, respectively, to make each circle fit best with the spherical shape of the lateral femoral condyles. The LFCI is defined as the ratio of the radius of the posterior circle (Rp) and the radius of the anterior circle (Ra): LFCI = Rp/Ra

LFCI was assessed on midsagittal MRI slice via the method proposed by Hodel et al. [12]. T1-weighted MRI coronal plane showing the most prominent popliteal groove (Fig. 1A) and the PD-FS transvers MRI plane showing the complete femoral trochlea with cartilage and intact posterior femoral condyles (Fig. 1B) were selected. The relevant T1-weighted midsagittal MRI slice of the lateral condyle was identified, and two circles were drawn to the cartilage margin of the anterior and posterior femoral condyles (Fig. 1C). LFCI is defined as the ratio between the radius of the posterior and anterior circle (Rp/Ra).

**Fig. 2:**
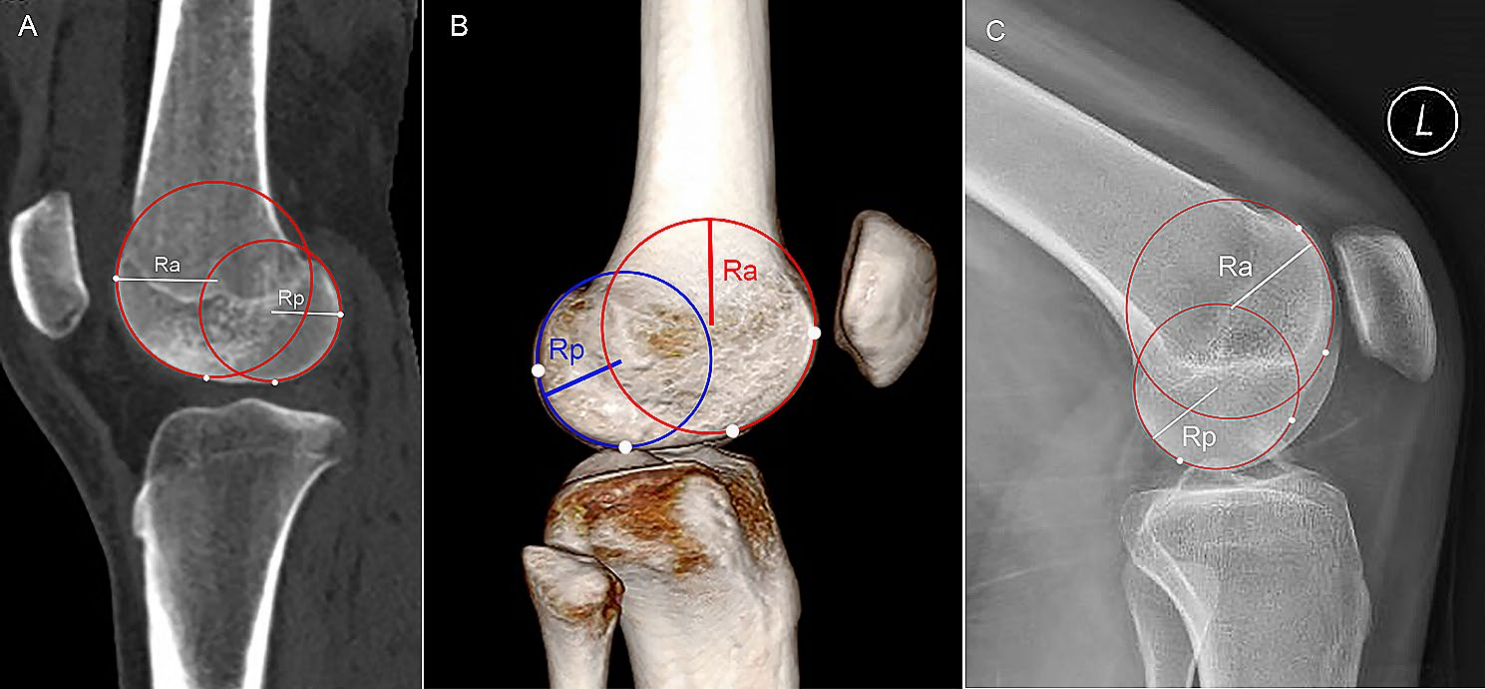
The measurement of lateral femoral condyle ratio (LFCR) by radiological data. **(A)** The midsagittal CT slice showing intact anterior and posterior femoral condyles is selected. **(B)** 3D–CT is reconstructed and standard lateral view is selected. **(C)** The lateral view of conventional radiograph is selected. Two circles are drawn to the bony edge and are congruent with the sphericity of the lateral femoral condyle. LFCI is calculated by a formula: LFCI = Rp/Ra

Sagittal reconstruction of the CT images was conducted via ADW4.6 workstation to reduplicate the LFCI measurement on MRI. The reconstructed midsagittal plane is parallel to the cortex of lateral femoral condyle with approximately 10–15 degrees of internal rotation in the anteroposterior direction. Two circles were drawn to the bony edge and were congruent with the sphericity of the LFC [20] (Fig. 2A). Three-dimensional CT (3D-CT) was reconstructed to make medial/posterior and lateral/posterior femoral condyles overlap (lateral view) via image post-processing techniques. Similar measurement method was performed to calculate the LFCI on 3D-CT (Fig. 2B). According to the method described by Vasta et al. [13], two circles fitting with the bony margin of LFC were drawn on the standard lateral view of CR to calculate LFCI (Fig. 2C).

#### LFCR

**Fig. 3:**
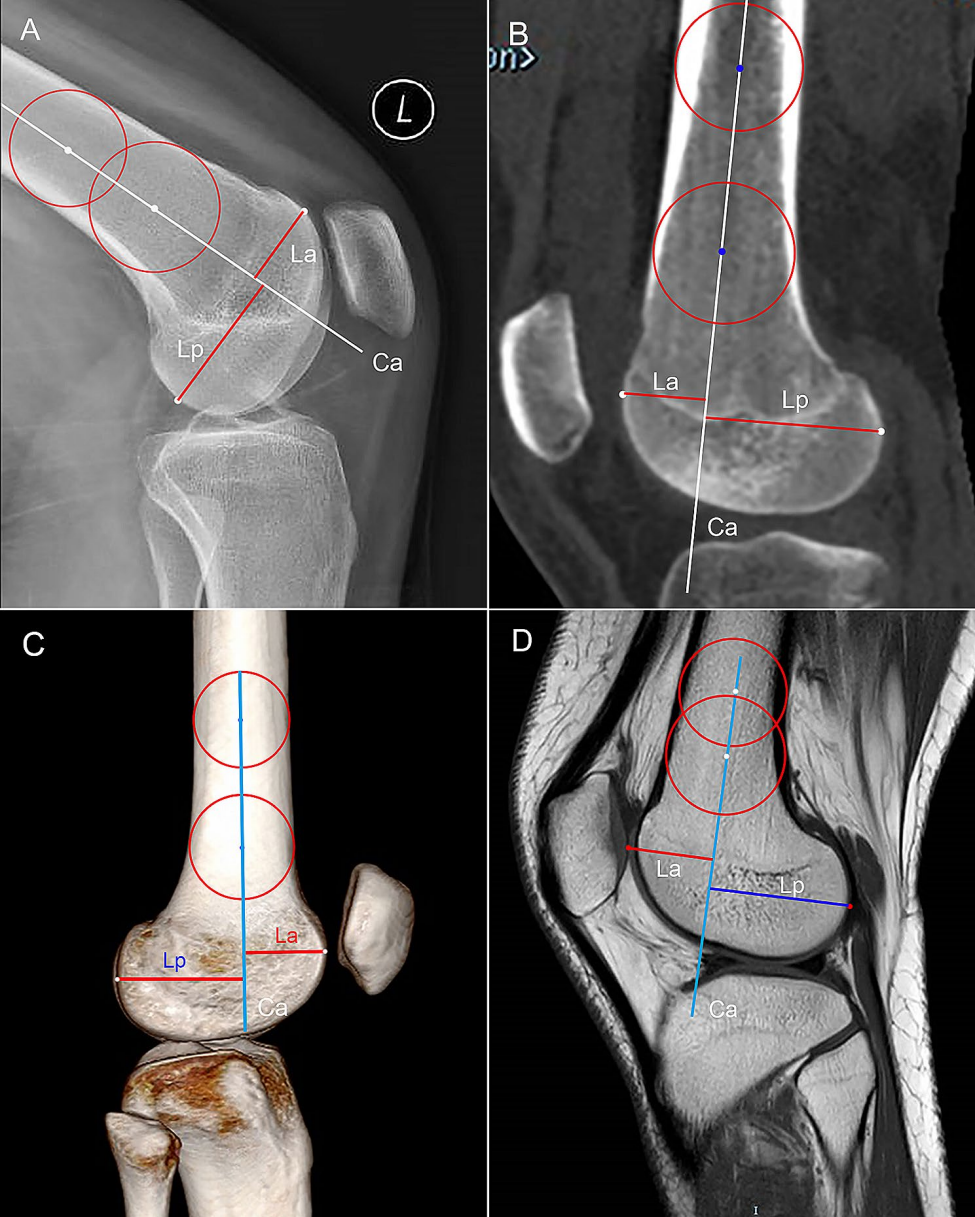
The measurement of lateral femoral condyle ratio (LFCI). **(A)** Lateral view of conventional radiograph is selected. Two circles are drawn to tangent with the anterior and posterior femur cortex, and the central longitudinal axis (Ca) passing through the centers of the two circles is identified. The vertical length between the most posterior or anterior portion of lateral femoral condyle and the Ca (Lp and La, respectively) is measured to calculate LFCI: Lp/La. **(B)** midsagittal CT slice is selected. **(C)** 3D–CT is reconstructed and standard lateral view is selected. **(D)** Midsagittal MRI slice is selected, and the Lp and La is the vertical distance between the cartilaginous margin (red dots) of femoral condyles and the Ca, respectively

Lateral view of CR was used to measure the LFCR according to the method reported previously [9]. The central longitudinal axis (Ca) of the distal femur was defined by drawing a proximal and a distal circle and connecting the centers of the two circles. The most anterior point and the most posterior point of the lateral condyle were marked to identify its vertical distance to the Ca (La and Lp, respectively). LFCR is the ratio of Lp/La (Fig. 3A). Likewise, the midsagittal CT slice showing intact LFC and complete distal femur shaft was reconstructed to identify the Ca of the distal femur. The most anterior and posterior points of LFC were marked to measure LFCR (Fig. 3B). Similar measurement method was performed for assessing the LFCR on 3D-CT (Fig. 3C).

LFCR measurement was performed on MRI in accordance with the method proposed by He et al. [21]. The Ca was the anatomic axis of the distal femur, and the most anterior and posterior points of the femoral cartilage were marked on midsagittal MRI slice of LFC to calculate its vertical distances to the Ca (Fig. 3D).

#### LCI

**Fig. 4:**
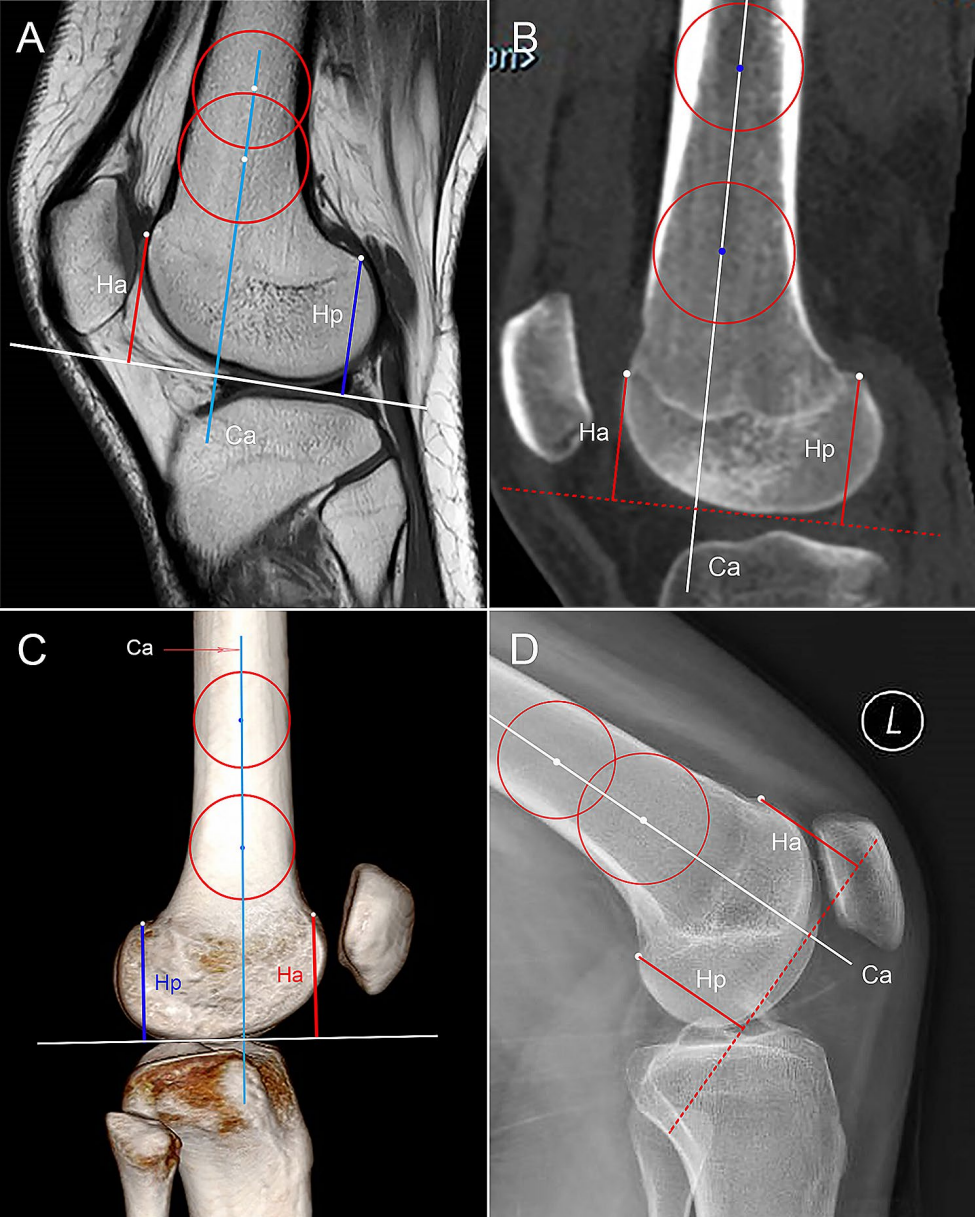
The measurement of lateral condyle index (LCI). **(A)** Midsagittal MRI slice is selected. Two circles tangent with the cortex of distal femur are drawn to identify the central longitudinal axis (Ca). The highest portion of anterior and posterior femoral condyle cartilage (white dots) are marked to measure to height of femoral condyles (Ha and Hp, respectively). LCI is defined as the ratio of Hp/Ha. Similarly, referring to the continuous portions of femur cortex and femoral condyle (white dots), the LCI is measured on sagittal CT slice **(B)**, on 3D–CT **(C)**, and on conventional radiograph **(D)** by similar methods

Biedert et al. [8] proposed a method for evaluating the LCI on midsagittal MRI plane of the lateral condyle. The Ca and a tangent line on the distal femoral condyle at 90° to the Ca were identified. The highest points of anterior and posterior femoral condylar cartilage were marked to measure the height of LFC in relation to the tangent line (Ha and Hp, respectively) (Fig. 4A). LCI is defined as the ratio of Hp/Ha in this study. The points where the anterior and posterior femoral cortex continued with the LFC were regarded as the reference points to measure the LCI on sagittal CT image, 3D-CT, and CR (Fig. 4B, C, and D).

#### LFCD

**Fig. 5:**
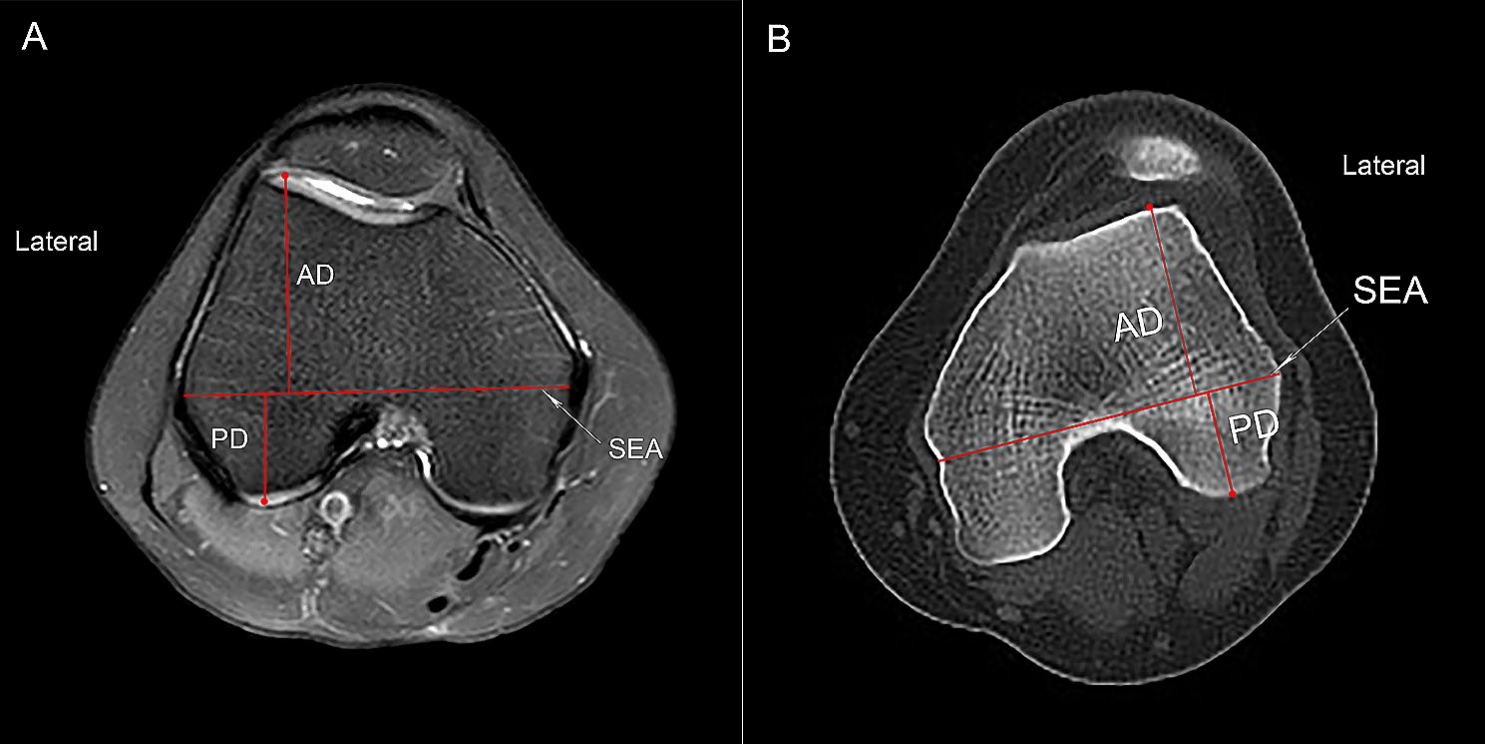
The measurement of lateral femoral condyle distance (LFCD). **(A)** The axial MRI slice showing the complete femoral trochlea with cartilage and intact posterior femoral condyles is selected. The surgical transepicondylar axis (SEA) are shown. The distance between the SEA and the posterior cartilaginous margin of the lateral condyle (PD) and the distance between the SEA and the anterior cartilaginous margin of the lateral condyle (AD) are measured. LFCD is defined as the ratio of PD/AD. **(B)** Similarly, the axial CT slice is selected. The length of anterior and posterior femoral condyles is measured to calculate LFCD

Referring to the method of Kobayashi et al. [11] and Geraghty et al. [22], the transverse MRI or CT plane showing the intact “Roman Arch” and femoral condyles was selected to measure the LFCD. The surgical transepicondylar axis (SEA) was defined as the line through the sulcus of the medial epicondyle and the prominence of the lateral epicondyle. The distance between the SEA and the posterior or the anterior margin of the LFC (PD and AD, respectively) were measured to calculate the LFCD (PD/AD) (Fig. 5).

### Statistical analysis

All the statistical analysis was performed by a well-trained orthopedist independently via the SPSS software (version 21.0; IBM Corp). The intra- and inter-observer reliability for each measurement was assessed by calculating intra-class correlation coefficient (ICC), with a value of more than 0.75 indicating good agreement, and a value of more than 0.90 indicating excellent agreement [23]. The Bland–Altman analysis and 95% limits of agreement (LOA) were performed [24] to further quantify the reliability of each measurement between different imaging modalities and between observers via GraphPad Software (version 8.0.2, San Diego, California USA). The ICC was also used to estimate the inter-modality reliability for LFCI, LFCR, LCI, and LFCD between MRI, sagittal CT image, CR, and 3D-CT. Distribution characteristic of the data was assessed by Shapiro–Wilk normality test. Pearson and spearman correlation analysis were conducted to identify the relationships among these parameters. A *P*-value of < 0.05 was considered statistically significant.

Power analysis was conducted via G-Power software (version 3.1.9.6, Universitat Dusseldorf, Germany). For the effect size of 0.41 according to the correlation between LFCI and LCI, a power of 0.98 was calculated (n, 73; alpha, 0.05).

## Results

A total of 73 patients (48 females and 25 males, mean age ± standard deviation 23.3 ± 6.2) with unilateral LPD were included in this study (Supplementary Fig. 1). According to the Dejour classification of trochlear dysplasia [25], 65 patients are with sever trochlear dysplasia (Type B-D). The ICC values for the intra-observer reliability of each measurement are shown in Table 1. Among the observers, the overall intra-observer reliability was highest for the radiologist and lowest for the junior orthopedist. The LFCD had an excellent intra-observer reliability on MRI (0.90–0.94) and on sagittal CT images (0.91–0.93). The ICC values for the intra-observer agreement of LCI were lower on CT and CR (ICCs < 0.75). The intra-observer reliability of the LFCI and LFCR on MRI, CT, and CR was good to excellent (0.76–0.94).

**Table 1:** Intra-observer reliability of the anatomic parameters, showing the ICC and 95% confidence interval

	MRI	CT	CR	3D–CT
Radiologist				
LFCI	0.93 (0.85,0.96)	0.82 (0.73,0.89)	0.85 (0.76,0.93)	0.94 (0.86,0.98)
LFCR	0.87 (0.81,0.92)	0.85 (0.79,0.93)	0.92 (0.86,0.96)	0.91 (0.83,0.95)
LCI	0.85 (0.74,0.93)	0.73 (0.65,0.88)	0.74 (0.62,0.84)	0.87 (0.76,0.94)
LFCD	0.94 (0.87,0.98)	0.93 (0.85,0.97)	–	–
Senior Orthopedist				
LFCI	0.89 (0.82,0.95)	0.81 (0.69,0.85)	0.83 (0.71,0.92)	0.91 (0.83,0.95)
LFCR	0.81 (0.74,0.88)	0.83 (0.72,0.91)	0.90 (0.82,0.96)	0.88 (0.79,0.94)
LCI	0.83 (0.72,0.89)	0.70 (0.59,0.84)	0.72 (0.68,0.83)	0.85 (0.78,0.90)
LFCD	0.91 (0.83,0.96)	0.92 (0.86,0.96)	–	–
Junior Orthopedist				
LFCI	0.87 (0.83,0.96)	0.81 (0.68,0.87)	0.78 (0.67,0.86)	0.86 (0.77,0.93)
LFCR	0.76 (0.68,0.87)	0.79 (0.69,0.85)	0.83 (0.75,0.91)	0.88 (0.75,0.94)
LCI	0.82 (0.74,0.91)	0.67 (0.53,0.81)	0.71 (0.61,0.85)	0.81 (0.69,0.87)
LFCD	0.90 (0.81,0.95)	0.91 (0.84,0.97)	–	–

LFCI, lateral femoral condyle index; LFCR, lateral femoral condyle ratio; LCI, lateral condyle index; LFCD, lateral femoral condyle distance; ICC, intra-class correlation coefficient; MRI, magnetic resonance imaging; CT, computed tomography (sagittal slice); CR, conventional radiograph; 3D–CT, three-dimensional CT.

The ICC values for the inter-observer reliability are shown in Table 2. The inter-observer reliability for the parameters between radiologist and junior orthopedist was lower than that between radiologist and senior orthopedist. Between radiologist and senior orthopedist, the inter-observer reliability of all the parameters was good for MRI, CT, and 3D–CT (ICC > 0.75). Parameters measured by 3D–CT and MRI had higher inter-observer agreement than by CT and CR. The inter-observer agreement for the LCI on CR was low, with an ICC of less than 0.75. The mean difference in each parameter between observers calculated by Bland–Altman analysis is shown in Table 3.

**Table 2:** Inter-observer reliability of each measurement, showing the ICC and 95% confidence interval

	LFCI	LFCR	LCI	LFCD
MRI				
RA/SO	0.89 (0.78,0.95)	0.85 (0.78–0.91)	0.83 (0.75–0.90)	0.88 (0.82–0.97)
RA/JO	0.79 (0.68–0.92)	0.74 (0.62–0.85)	0.74 (0.69–0.84)	0.89 (0.84–0.98)
CT				
RA/SO	0.82 (0.71–0.90)	0.84 (0.72–0.93)	0.75 (0.64–0.88)	0.87 (0.82–0.93)
RA/JO	0.72 (0.57–0.86)	0.73 (0.60–0.85)	0.67 (0.55–0.82)	0.85 (0.77–0.91)
CR				
RA/SO	0.78 (0.68–0.87)	0.82 (0.74–0.91)	0.67 (0.53–0.82)	–
RA/JO	0.68 (0.55,0.83)	0.70 (0.59–0.87)	0.68 (0.56–0.84)	–
3D–CT				
RA/SO	0.86 (0.75,0.91)	0.88 (0.81,0.93)	0.81 (0.67,0.89)	–
RA/JO	0.82 (0.73,0.88)	0.83 (0.72,0.91)	0.77 (0.68,0.85)	–

LFCI, lateral femoral condyle index; LFCR, lateral femoral condyle ratio; LCI, lateral condyle index; LFCD, lateral femoral condyle distance; ICC, intra-class correlation coefficient; MRI, magnetic resonance imaging; CT, computed tomography (sagittal slice); CR, conventional radiograph; 3D–CT, three-dimensional CT; RA, radiologist; SO, senior orthopedist; JO, junior orthopedist

**Table 3:** Bland–Altman analysis to evaluate the reliability of each parameter between observers, showing the mean difference of each parameter and 95% limits of agreement

	LFCI	LFCR	LCI	LFCD
MRI				
RA vs. SO	0.053 (0.142)	0.068 (0.154)	0.117 (0.286)	0.048 (0.103)
RA vs. JO	0.062 (0.167)	0.076 (0.149)	0.128 (0.303)	0.051 (0.112)
SO vs. JO	0.064 (0.185)	0.095 (0.203)	0.122 (0.294)	0.062 (0.107)
CT				
RA vs. SO	0.062 (0.148)	0.075 (0.163)	0.122 (0.274)	0.053 (0.112)
RA vs. JO	0.073 (0.166)	0.084 (0.152)	0.131 (0.286)	0.047 (0.129)
SO vs. JO	0.067 (0.179)	0.094 (0.197)	0.126 (0.269)	0.071 (0.118)
CR				
RA vs. SO	0.058 (0.157)	0.055 (0.174)	0.120 (0.288)	–
RA vs. JO	0.072 (0.161)	0.080 (0.153)	0.126 (0.294)	–
SO vs. JO	0.070 (0.182)	0.104 (0.223)	0.124 (0.275)	–
3D–CT				
RA vs. SO	0.047 (0.133)	0.065 (0.152)	0.118 (0.257)	–
RA vs. JO	0.066 (0.148)	0.078 (0.147)	0.127 (0.284)	–
SO vs. JO	0.064 (0.162)	0.089 (0.192)	0.125 (0.288)	–

LFCI, lateral femoral condyle index; LFCR, lateral femoral condyle ratio; LCI, lateral condyle index; LFCD, lateral femoral condyle distance; MRI, magnetic resonance imaging; CT, computed tomography (sagittal slice); CR, conventional radiograph; 3D–CT, three-dimensional CT; RA, radiologist; SO, senior orthopedist; JO, junior orthopedist

Table 4 shows the ICC values for the reliability of each measurement between different imaging modalities by using the measurements of radiologist and senior orthopedist, because these had a higher intra-observer reliability. All the parameters had good reliability between MRI and 3D–CT or between MRI and CT (ICC > 0.75). LFCI, LFCR, and LCI showed a moderate agreement between MRI and CR or between CT and CR (ICC < 0.75). Bland–Altman analysis by using the measurement of the radiologist showed that the mean difference of the LFCI was 0.048 (LOA 0.152) for MRI versus CT, 0.059 (LOA 0.154) for MRI versus CR, 0.057 (LOA 0.162) for CT versus CR, and 0.045 (LOA 0.147) for MRI versus 3D–CT.

**Table 4:** Inter-modality reliability, showing the ICC and 95% confidence interval

	MRI vs. CT	MRI vs. CR	CT vs. CR	MRI vs. 3D–CT
LFCI				
Radiologist	0.82 (0.72,0.89)	0.73 (0.57,0.83)	0.70 (0.52,0.81)	0.88 (0.81,0.96)
Senior Orthopedist	0.76 (0.65,0.87)	0.69 (0.52,0.84)	0.66 (0.52,0.86)	0.89 (0.78,0.95)
LFCR				
Radiologist	0.78 (0.61,0.86)	0.72 (0.53,0.85)	0.68 (0.56,0.89)	0.81 (0.72,0.87)
Senior Orthopedist	0.80 (0.66,0.91)	0.74 (0.55,0.86)	0.65 (0.49,0.73)	0.77 (0.68,0.85)
LCI				
Radiologist	0.77 (0.56,0.85)	0.68 (0.48,0.76)	0.65 (0.46,0.75)	0.76 (0.59,0.87)
Senior Orthopedist	0.79 (0.61,0.90)	0.70 (0.51,0.82)	0.66 (0.49,0.76)	0.77 (0.62,0.85)
LFCD				
Radiologist	0.85 (0.74,0.92)	–	–	–
Senior Orthopedist	0.87 (0.78,0.96)	–	–	–

LFCI, lateral femoral condyle index; LFCR, lateral femoral condyle ratio; LCI, lateral condyle index; LFCD, lateral femoral condyle distance; ICC, intra-class correlation coefficient; MRI, magnetic resonance imaging; CT, computed tomography (sagittal slice); CR, conventional radiograph; 3D–CT, three-dimensional CT.

Because good intra- and inter-observer reliability was shown, the average value of each parameter measured by the radiologist and senior orthopedist on MRI was used to conduct correlation analysis. Except for the LFCR, all the parameters measured by MRI were conformed to normal distribution. The LFCI was correlated with LFCR (ρ = 0.422, *P* = 0.022), LCI (*r* = 0.413, *P* = 0.037), and LFCD (*r* = 0.459, *P* = 0.014). The correlation between the LCI and LFCR (ρ = 0.137) or LFCD (*r* = 0.153) was not significant (*P* > 0.05). Similar results were shown regarding the parameters measured on 3D–CT (Table 5).

**Table 5:** Correlations among the parameters measured by MRI and 3D–CT, showing the correlation coefficients

	MRI				3D–CT		
	LFCI	LFCR ^a^	LCI		LFCI	LFCR ^a^	LCI
LFCI	-	0.422*	0.413*		-	0.453*	0.397*
LFCR ^a^	0.422*	-	0.137		0.453*	-	0.167
LCI	0.413*	0.137	-		0.397*	0.167	-
LFCD	0.459*	0.554*	0.153		-	-	-

LFCI, lateral femoral condyle index; LFCR, lateral femoral condyle ratio; LCI, lateral condyle index; LFCD, lateral femoral condyle distance; MRI, magnetic resonance imaging; 3D–CT, three-dimensional computed tomography; ^a^, the results of spearman correlation analysis; *, statistical significance *P* < 0.05

## Discussion

The most important finding of this study was that the intra- and inter-observer reliability for the LFCI was good to excellent on MRI and 3D–CT with the lowest mean difference between observers, and the ICC value of the LFCI measurement for MRI versus 3D–CT showed better agreement. The LFCI was correlated with the height (LCI) and length (LFCR and LFCD) of LFC and could serve as a comprehensive measurement to quantify the morphology of LFC.

Patients with LPD are often accompanied by a variety of skeletal malformations, among which the abnormal morphology of LFC, such as asymmetry of anterior and posterior condyles, is regarded as a significant risk factor [4, 7]. Numerous measurement methods and imaging modalities have been used to evaluate the morphology of LFC [12, 27]. However, there is limited literature on LFC morphology in LPD patients, and further research is needed to determine the most suitable measurement or imaging method in clinical practice.

It is accepted that a substantial experience in radiological measurement is responsible for a high intra- or inter-observer reliability [28]. In this study, even though the junior orthopedist was trained for the measurement methods, the ICC values of different parameters for the intra- and inter-observer reliability were lower than those of the radiologist and senior orthopedist. Due to young doctors’ limited clinical experience, unfamiliarity with software, inadequate understanding of anatomy, and suboptimal layer selection, there may be a slight decrease in measurement accuracy despite their professional training (although the ICC value remains within an acceptable range). To the best of our knowledge, this study is the first to verify the intra- and inter-observer reliability of different measurement methods between MRI, CT, CR, and 3D–CT for quantifying the morphology of LFC.

The LFCI could reliably reflect the morphology of LFC but has not been researched in patients with LPD. Li et al. [29] performed the LFCI measurement on MRI and CR but did not report the ICC values. Vasta et al. [13] measured the LFCI on CR without reporting the intra- or inter-observer reliability. In our study, the ICC of the LFCI measured on CR was 0.85 for intra-observer reliability and 0.78 for inter-observer reliability. In a study by Nowak et al. [30], the LFCI measurement was performed on MRI by a single investigator, but the intra-observer reliability was not elucidated. Hodel et al. [12] found that the ICC of the LFCI measured by MRI was 0.96 for the intra-observer reliability and 0.89 for the inter-observer reliability, compared to 0.93 and 0.89 in our study, respectively. Hodel et al. [20] reported that the LFCI measurement on sagittal CT images had a good inter-observer reliability (ICC > 0.77). In our study, we found a moderate (ICC = 0.72) inter-observer agreement between radiologist and junior orthopedist, which may attribute to the complicated process of sagittal reconstruction and trochlear dysplasia. Micicoi et al. [31] performed the LFCI measurement on 3D–CT but did not reveal the reliability of the method. In our study, excellent intra-observer reliability (ICC = 0.94) and good inter-observer reliability (ICC = 0.86) were identified, indicating that 3D–CT could be an appropriate alternative to reliably measure the LFCI in patients with LPD.

The Bland–Altman analysis is utilized to evaluate the mean difference between measurement methods and calculates 95% LOA [32]. When looking at the results of the LFCI between the radiologist and senior orthopedist, the largest difference was 0.062 for sagittal CT images, but lower for 3D–CT (0.047) and MRI (0.053). In addition, the reliability of the LFCI measurement for MRI versus CT showed better agreements, with a mean difference of 0.045–0.048. Patients may suffer various degrees of trochlear dysplasia, but the LFCI measured on MRI and 3D–CT is reliable.

LFCR serves as a parameter for evaluating the length of LFC in the anteroposterior direction. He et al. [21] conducted the LFCR measurement on MRI and revealed an ICC of 0.83 and of 0.85 for intra- and inter-observer reliability, respectively. In our study, the intra- and inter-observer reliability of the LFCR on MRI was 0.87 and 0.85, respectively. Jeon et al. [33] elucidated that the intra-observer reliability of the LFCR measurement on CR was 0.82 (95% CI 0.73–0.88). Kim et al. [27] reported that the ICC values of the LFCR on CR showed good intra- and inter-observer agreements, compared to 0.92 and 0.82 in our study, respectively. To the best of our knowledge, CT was used to evaluate the LFCR for the first time. We found that the intra- and inter-observer reliability was 0.91 and 0.88 for 3D–CT, compared to 0.85 and 0.84 for sagittal CT images, respectively.

The Bland–Altman analysis showed that the mean difference between the radiologist and the senior orthopedist was lowest for CR (0.055) and highest for sagittal CT images (0.075). The ICC showed highest agreement for MRI versus 3D–CT and lowest agreement for sagittal CT images versus CR. 3DCT images are easily created by post-processing technology, are independent of patient knee exion, making it convenient for precise localization. With consistent methodology, the measured data will exhibit high repeatability. On the other hand, CR has a low resolution and is not suitable for complex image post-processing technology. In addition, it also depends on the position of the patient at the time of examination, which leads to difficulties for the measurement of special anatomical structures to some extent. These results indicated that CR and 3D–CT served as appropriate modalities for measuring LFCR and evaluating the length of LFC in patients with LPD.

With regard to another parameter reflecting the length of LFC, Roger et al. [34] found an excellent inter-observer reliability for the LFCD measured on axial CT images. Furthermore, Yang et al. [7] reported that in patients with patellar dislocation, the ICC values of the LFCD measured on axial CT images for inter-observer reliability ranged from 0.83 to 0.98. Geraghty et al. [22] performed the length measurement on axial MRI plane in patellar dislocators, and excellent intra- and inter-observer agreements were shown. In our study, axial MRI and CT slices had good to excellent intra- and inter-observer reliability for the LFCD measurement. Good agreement in the LFCD measurement for MRI versus CT was found as well. These results indicated that the LFCD measured by both MRI and CT could reliably reflect the anterior and posterior length of LFC.

Biedert et al. [8] performed the LCI measurement on MRI to evaluate the height of LFC in patients with patellar dislocation and found an excellent inter-observer agreement for the method. Ismailidis et al. [35] also utilized MRI to measure the LCI but did not report the reliability. In our study, we conducted the measurement on different imaging modalities and found good intra- and inter-observer reliability for MRI and 3D–CT. With regard to the reliably between different imaging modalities, the ICC values showed good agreements for MRI versus CT and for MRI versus 3D–CT. These results indicated that MRI and 3D–CT could clinically serve as better modalities for measuring LCI and assessing the height of LFC.

Correlation analysis showed that the LFCI was significantly correlated with the LCI, LFCR, and LFCD, indicating that the LFCI could to some extent reflect both the height and length of LFC. On the other hand, it should be noted that each imaging modality has its advantages. MRI has a strong ability of recognizing soft tissues without radiation exposure risk but is expensive with a limited scan range. CT scan is much more detailed for bony structures, but excessive radiation exposure happens and image post-processing is relatively complicated. CR is more convenient but has lower resolution and strict posture requirement of the knee when conducting examination [36]. Clinically, a comprehensive consideration should be given to the measurement method for evaluating the morphology of LFC, including the measurement reliability, the experience of the investigators, and the advantage of the imaging modality.

Our study had some limitations. First, control group with healthy individuals was not included in this study, and it is unclear whether lateral condyle dysplasia occurred in patients with LPD, which warrants further investigation. Second, the LFCD was not measured on 3D–CT in this study, because it is difficult to identify the SEA on 3D model of femoral condyles [37]. A study regarding this issue is necessary in the future. Third, lateral condyle width was not studied, and the parameter that could comprehensively reflect the height/length/width of lateral femoral condyle has not been revealed. Fourth, whether the morphology of LFC is related to coronal malalignment of lower limb in patients with LPD is worthy of further study. Fifth, the influence of trochlear dysplasia to the measurement methods is not detailed in this study, but the overall reliability of the LFCI measurement is good in patients with LPD. Sixth, the tube voltage and current levels of the CT examination are relatively high but within acceptable range [26], which we think would not influence the measurement accuracy of this study.

Along with previous literature and our findings, patients with LPD exhibit various deformities such as trochlear dysplasia, femoral anteversion, increased TT-TG distance, and abnormal development of the posterolateral condyle of the femur. However, researchers have only recently started to pay serious attention to measuring the lateral condyle in cases of LPD. The optimal measurement method and imaging technique that can accurately reflect lateral condyle development remain unclear. This is crucial because surgical interventions rely on precise measurements of patient deformities. Our objective is to identify more suitable measurement methods and imaging modalities that can assist clinicians in accurately evaluating the lateral condyle for patients with LPD.

## Conclusion

The LFCI could be reliably measured by MRI and 3D–CT. The LFCI was associated with both the height and length of LFC and could serve as a comprehensive parameter for quantifying the morphology of LFC in patients with LPD.

### Electronic supplementary material

Below is the link to the electronic supplementary material.

Supplementary Material 1. Supplementary Fig. 1. Flow chart of inclusion and exclusion criteria. LPD, lateral patellar dislocation; MRI, magnetic resonance image; CR, conventional radiograph; CT, computed tomography.

## Data Availability

The data associated with the paper is not publicly available but is available from the corresponding author upon reasonable request.

## Abbreviations

LFC: Lateral femoral condyle
LPD: Lateral patellar dislocation
CR: Conventional radiograph
3D–CT: Three-dimensional CT
LFCI: The lateral femoral condyle index
LFCR: Lateral femoral condyle ratio
LFCD: Lateral femoral condyle distance
