# Supplementary material for: Evaluating the reliability of the lateral femoral condyle measuring methods by different modalities for patients with lateral patellar dislocation
Source: BMC Musculoskelet Disord. 2024 May 18;25:388. doi: 10.1186/s12891-024-07495-x (PMC11102213; doi:10.1186/s12891-024-07495-x)
Supplement: Supplementary file 1 — Supplementary Material 1. Supplementary Fig. 1. Flow chart of inclusion and exclusion criteria. LPD, lateral patellar dislocation; MRI, magnetic resonance image; CR, conventional radiograph; CT, computed tomography. [file 12891_2024_7495_MOESM1_ESM.pdf]

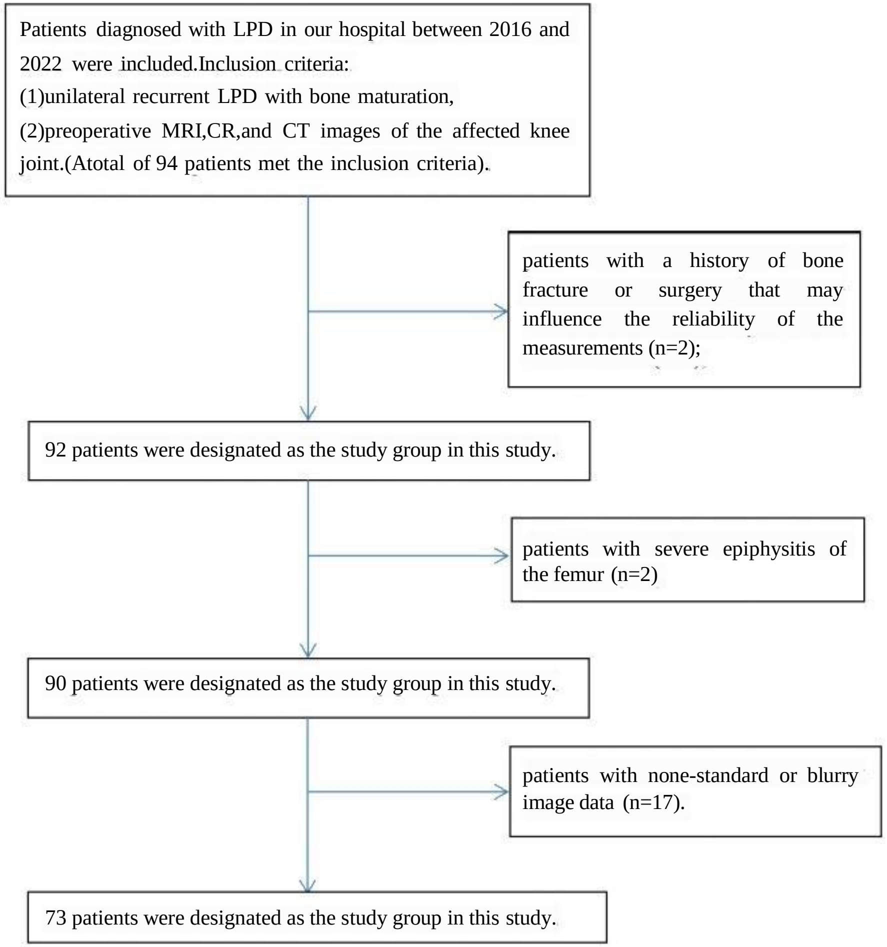

Supplementary Fig 1.

Flow chart of inclusion and exclusion criteria. LPD, lateral patellar dislocation; MRI, magnetic resonance image; CR, conventional radiograph; CT, computed tomography.
